# Photoreactivation Activities of Rad5, Rad16A and Rad16B Help *Beauveria bassiana* to Recover from Solar Ultraviolet Damage

**DOI:** 10.3390/jof10060420

**Published:** 2024-06-13

**Authors:** Xin-Cheng Luo, Lei Yu, Si-Yuan Xu, Sheng-Hua Ying, Ming-Guang Feng

**Affiliations:** Institute of Microbiology, College of Life Sciences, Zhejiang University, Hangzhou 310058, China; 12207006@zju.edu.cn (X.-C.L.); 11907037@zju.edu.cn (L.Y.); 12007036@zju.edu.cn (S.-Y.X.); yingsh@zju.edu.cn (S.-H.Y.)

**Keywords:** fungal insecticides, solar UV damage, anti-UV proteins, DNA photorepair index, nucleotide excision repair index

## Abstract

In budding yeast, Rad5 and Rad7-Rad16 play respective roles in the error-free post-replication repair and nucleotide excision repair of ultraviolet-induced DNA damage; however, their homologs have not yet been studied in non-yeast fungi. In the fungus *Beauveria bassiana*, a deficiency in the Rad7 homolog, Rad5 ortholog and two Rad16 paralogs (Rad16A/B) instituted an ability to help the insect-pathogenic fungus to recover from solar UVB damage through photoreactivation. The fungal lifecycle-related phenotypes were not altered in the absence of *rad5*, *rad16A* or *rad16B,* while severe defects in growth and conidiation were caused by the double deletion of *rad16A* and *rad16B*. Compared with the wild-type and complemented strains, the mutants showed differentially reduced activities regarding the resilience of UVB-impaired conidia at 25 °C through a 12-h incubation in a regime of visible light plus dark (L/D 3:9 h or 5:7 h for photoreactivation) or of full darkness (dark reactivation) mimicking a natural nighttime. The estimates of the median lethal UVB dose LD_50_ from the dark and L/D treatments revealed greater activities of Rad5 and Rad16B than of Rad16A and additive activities of Rad16A and Rad16B in either NER-dependent dark reactivation or photorepair-dependent photoreactivation. However, their dark reactivation activities were limited to recovering low UVB dose-impaired conidia but were unable to recover conidia impaired by sublethal and lethal UVB doses as did their photoreactivation activities at L/D 3:9 or 5:7, unless the night/dark time was doubled or further prolonged. Therefore, the anti-UV effects of Rad5, Rad16A and Rad16B in *B. bassiana* depend primarily on photoreactivation and are mechanistically distinct from those for their yeast homologs.

## 1. Introduction

Solar ultraviolet (UV) irradiation provides a ubiquitous stress to filamentous fungi associated with plants and arthropods on the Earth’s surface. Fungal anti-UV activity is exploitable for the optimal application strategies of fungal insecticides and acaricides against arthropod pests on sunny days of summer [1]. Strong sunlight comprises wavelengths of UVB (290–320 nm) as a main source of DNA damage and UVA (320–400 nm) as an inducer of reactive oxygen species or oxidative stress [2,3]. As active agents of mycopesticides, formulated conidia are highly sensitive to shorter-wavelength UVB irradiation [4,5,6], making UVB a major source of solar UV damage to conidia applied for pest control. Eukaryotic DNA damage induced by UV features cytotoxic DNA photoproducts formed by covalent linkages, which can be reversed by nucleotide excision repair (NER) and photorepair [7,8,9,10,11]. However, the molecular mechanisms underlying the recovery of filamentous fungal cells from solar UV damage are still only minimally understood.

NER is a slow process that occurs independent of light and has been intensively studied in *Saccharomyces cerevisiae*. In earlier studies, a huge family of anti-UV radiation (RAD) proteins and partners have been identified as NER factors [12]. In the yeast, such factors form an array of protein complexes through multiple protein–protein interactions, and the complexes act as endonucleases, helicases, ligases and polymerases in the processes of transcription-coupled or global-genome NER (TC-NER or GG-NER) and/or of error-free post-replication repair (PRR) for the bypass of DNA damage [13,14,15,16,17], as reviewed in [18]. For example, the Rad4-Rad23-Rad33 complex can recognize impaired DNA to initiate GG-NER [15,19,20]. The Rad1-Rad10 complex is an endonuclease needed for GG-NER [21,22,23,24,25]. Rad2 and Rad1-Rad10, crucial for DNA damage incisions [26,27], are components of a large protein complex consisting of Rad14, eight subunits of TFIIH (NER-required transcription factor) and the replication protein RPA [28]. Rad7 and Rad16 can remove DNA lesions from the non-transcribed strand of active genes [29] and interact with each other to form a stable complex, which binds specifically to DNA lesions [30], stimulates the dual cleavage of UV-irradiated DNA in vitro [31], and hence is required for GG-NER [32,33]. Rad16 triggers histone H3 acetylation under UV irradiation and takes part in NER at silenced loci though the induction of H3 methylation [34,35]. A combination of Rad4-Rad23 and Rad7-Rad16 can enhance the binding activity to UV-damaged DNA [31]. Rad7-Rad16 interacts not only with Rad4 but also with the Elc1-Cul3 complex, forming a large, cullin-containing E3 ubiquitin ligase needed for the UV-reliant ubiquitination of Rad4 and other proteins associated with chromatin [36,37]. Rad26 linked to RNA polymerase II has ATPase activity needed for TC-NER and the bypass of DNA damage [38,39], and its phosphorylation can increase the TC-NER rate of DNA damage [40]. The E3 ubiquitin ligase Rad5 interacts with Rad18 and Ubc13 to form a large protein complex composed of Rad5, Rad6-Rad18 and Ubc13-Mms2 as the central players in error-free PRR [41,42,43]. Together, these studies have unveiled that the anti-UV roles of the mentioned RAD proteins and partners depend on their activities in the yeast GG-NER, TC-NER and error-free PRR.

In contrast, photorepair is a process that rapidly repairs shorter UV-caused DNA damage under longer UV or white (visible) light [9,44,45]. In filamentous fungi, only one or two photolyases demonstrate photorepair activity, namely, Phr1 that repairs the DNA lesions cyclobutane pyrimidine dimers (CPDs) and/or Phr2 that repairs the DNA lesions (6–4)-pyrimidine-pyrimidone (6–4PP). This activity is in contrast with one or two cryptochromes that are classified to the same photolyase–cryptochrome family (PCF) but have no photorepair activity [46,47,48,49]. Filamentous fungi possess one to four PCF members that share a DNA_Photolyase domain determinant of photorepair activity. Why photorepair is dependent on photolyase(s) but independent of cryptochrome(s) is unclear until the nuclear localization of a PCF member has proved to be a prerequisite for photorepair activity. In *Beauveria bassiana*, nucleus-specific Phr1 and Phr2 exhibit photorepair activities against UVB-induced CPD and 6–4PP lesions, respectively, while the unique cryptochrome CryD localized in the cytoplasm lacks such an activity [50]. This prerequisite is linked to the high-probability presence of a nuclear localization signal (NLS) motif in the protein sequences of the photolyases instead of cryptochromes surveyed in fungal genomes [51].

Filamentous fungi possess homologs of those anti-UV RAD proteins characterized in the model yeast. However, such homologs had not been investigated until recent studies shed light upon the high photoreactivation activities of several homologs in the recovery of insect-pathogenic fungi from UVB damage. In *B. bassiana*, the Rad23 ortholog interacts with Phr2 and can largely recover the viability of conidia irradiated at a lethal UVB dose through 24 h of incubation at the regime of 3:21 h (L/D) rather than in full darkness [52]. WC1 and WC2 are two white-collar proteins confirmed as photolyase regulators in *B. bassiana* and *Metarhizium robertsii* and display high photorepair activities, in fact, much higher activities in photoreactivation than Phr1 and Phr2, although they lack a domain needed for photorepair activity [53,54,55]. The interactions of Rad1-WC2 and Rad10-Phr1 in *M. robertsii* and of either Rad1 or Rad10 with both WC1 and WC2 in *B. bassiana* confer very high photoreactivation activities on Rad1 and Rad10 in the two insect mycopathogens [54,56]. Unlike the Rad4-Rad23-Rad33 complex in the yeast, the insect pathogens have two Rad4 paralogs (Rad4A/B) but lack Rad33. An interaction between Rad4A and Rad23 endows them with high UVB resistance but different activities in the photoreactivation of UVB-impaired conidia despite the functional redundancy of Rad4B in *B. bassiana* and *M. robertsii* [57,58]. Rad2, Rad14 and Rad26 orthologs also display extraordinarily high photoreactivation activities in the insect pathogens due to the interactions of each with WC1, WC2, Rad1 and/or Rad10 in *M. robertsii* [59], and with WC1, WC2, Rad10 and RFA1 (a subunit of the DNA-binding protein, RFA) in *B. bassiana* [60]. The RAD homologs that have been characterized in recent studies also have an extant NER activity in the 24-h dark reactivation of low UVB dose-impaired conidia. However, their NER activities in dark reactivation are much lower than their photorepair activities in photoreactivation and are unable to recover conidia irradiated at sublethal/lethal UVB doses under the dark conditions mimicking night on the Earth’s surface. These progresses have largely expanded on a molecular basis for the filamentous fungal adaptation and resistance to solar UV, leading to a hypothesis that fungal photoreactivation is mechanistically far more complicated than the previously understood process dependent on only one or two photolyases [51]. In the insect pathogens, the photoreactivation and dark reactivation rates of the UVB-impaired or inactivated conidia used in the studies are dependable and easily accessible indices for the respective activities of photorepair and NER, which can also be directly quantified at a much higher cost than the indices.

To date, the anti-UV RAD homologs characterized in insect-pathogenic fungi are still very limited in comparison to dozens of RAD proteins and partners in the yeast. Rad7-Rad16 and Rad5 required for the respective processes of GG-NER and error-free PRR in the yeast [18] remain unknown in function. To close a knowledge gap, this study seeks to elucidate anti-UV effects of Rad5 ortholog and two Rad16 paralogs (Rad16A and Rad16B) in *B. bassiana*, which lacks a Rad7 homolog. We place an emphasis on whether their anti-UV effects depend on the NER-dependent dark reactivation or the photorepair-dependent photoreactivation of fungal conidia impaired by UVB radiation to different degrees. The results reinforce that the anti-UV effects of Rad5, Rad16A and Rad16B depend much more on photoreactivation than on dark reactivation on the Earth’s surface.

## 2. Materials and Methods

### 2.1. Microbial Strains and Media

The microbial strains and media used in this study are listed in Table 1.

### 2.2. Bioinformatic Analysis of Rad5 and Rad16 Homologs in Ascomycetes

The Rad5 (NP_013132, 1169 aa) and Rad16 (NP_009672, 790 aa) sequences of *S. cerevisiae* were used as queries to search online (http://blast.ncbi.nlm.nih.gov/Blast.cgi/, accessed on 11 June 2024) in the NCBI protein databases of selected ascomycetes including *B. bassiana*. The Rad5 or Rad16 homologs located in the databases were clustered to phylogenetic clades using the maximum likelihood method in the online program MEGA11 (http://www.megasoftware.net/, accessed on 11 June 2024). SMART analysis (http://smart.embl-heidelberg.de/, accessed on 11 June 2024) was performed to predict conserved domains from the amino acid sequences of each yeast query and the corresponding *B. bassiana* homolog for comparison. An NLS motif was predicted from the amino acid sequence of each yeast query or its *B. bassiana* homolog with a maximal probability at https://www.novopro.cn/tools/nls-signal-prediction (accessed on 11 June 2024).

### 2.3. Subcellular Localization of Rad5, Rad16A and Rad16B in B. bassiana

The fusion proteins Rad5-GFP, Raf16A-GFP and Rad16B-GFP were expressed in the WT strain using pAN52-GFP-bar as a backbone vector driven by the endogenous promoter P*tef1*. The coding regions of *rad5*, *rad16A* or *rad16B* were amplified from the WT cDNA using the primers in Table S1 and one-step cloning kit (Vazyme, Nanjin, China), followed by the fusion of each to *GFP* at 5′-terminus in the linearized vector. After verification with sequencing, each vector was transformed into WT using a method of transformation mediated by *A. tumefaciens* AGL-1 [61]. The resistance of *bar* to phosphinothricin (200 μg/mL) was employed for the screening of putative transgenic colonies. A transgenic strain showing a desirable green fluorescence signal was selected from the colonies generated by each transformation and incubated for 7 days on SDAY at an optimal regime of 25 °C and 12:12 (L/D) photoperiod. The conidia collected from the culture were suspended in SDBY (i.e., agar-free SDAY), followed by 2 days of shaking incubation at 25 °C and 150 rpm. Hyphae collected from the liquid culture were stained with a nucleus-specific dye (4.16 mM of 4′,6′-diamidine-2′-phenylindole dihydrochloride (DAPI)), followed by visualization for green (expressed) and red (stained) fluorescence signals of each fusion protein under a laser scanning confocal microscope at the excitation/emission wavelengths of 358/460 and 488/507 nm, respectively. The online program ImageJ (https://imagej.nih.gov/ij/, accessed on 11 June 2024) was adopted to quantify the ratios of nuclear versus cytoplasmic green fluorescence intensity (N/C-GFI) of each fusion protein from 15 cells of hyphae.

### 2.4. Y2H Assays for Protein–Protein Interactions

To explore potential factors to interact with Rad5, Rad16A or Rad16B from photolyases; photolyase regulators; and some other anti-UV proteins with high photoreactivation activities, Y2H assays were carried out as described previously [56,57,58,59,60]. In brief, the open reading frames of *rad5*, *rad16A*, *rad16B*, *phr1*, *phr2*, *wc1*, *wc2*, *rad1*, *rad10*, *rad2*, *rad6*, *rad18*, *rad14*, *rad4A*, *rad23*, *mms2*, *elc1*, *cul3A* and *cul3B* (see Table S1 for tag loci and used primers) were cloned from the WT cDNA as aforementioned, followed by the ligation of each one to pGADT7 (prey vector, AD) and pGBKT7 (bait vector, BD), respectively. The constructed AD and BD vectors were verified using sequencing and transformed into the respective Y187 and Y2HGold strains of *S. cerevisiae*, followed by 24 h of incubation on YPD for pairwise yeast mating at 30 °C. All yeast diploids were simultaneously screened with AD-LargeT-BD-P53 as a positive control and empty AD-BD and semi-empty constructs as the negative controls on double-dropout (SDM/-Leu/-Trp/X-α-Gal/AbA) and quadruple-dropout (SDM/-Leu/-Trp/-Ade/-His/X-α-Gal/AbA) plates. All yeast colonies were initiated via spotting 5 × 10^2^, 5 × 10^3^ and 5 × 10^4^ cells on the plates, followed by 3 days of incubation at 30 °C for photographing.

### 2.5. Generation of rad5, rad16A and rad16B Mutants

The genes *rad5*, *rad16A* and *rad16B* were disrupted by the deletion from the WT genome of a partial promoter/coding fragment of *rad5* (2350 bp; Figure S1A), a coding fragment of *rad16A* (1995 bp; Figure S1B), and the partial flanking and full-length coding regions of *rad16B* (4091 bp; Figure S1C) through the homologous recombination of the vector p0380-5′*x*-bar-3′*x*, in which *x* denoted a target gene to be disrupted. An identified Δ*rad5* or Δ*rad16B* mutant was successfully complemented with flanking and full-length coding regions of *rad5* (4853 bp) or *rad16B* (4515 bp) through the ectopic integration of p0380-*x*-sur. Using the same or modified strategies, the complementation of *rad16A* into its deletion mutant was unsuccessful over many attempts. The double deletion of *rad16A* and *rad16B* was achieved via the homologous recombination of p0380-5′rad16A-sur-3′rad16A to delete the 1995-bp coding fragment of *rad16A* from the identified Δ*rad16B* mutant (Figure S1D). The constructed deletion or complementation vectors were integrated into the WT or Δ*x* strain through transformation mediated via *A. tumefaciens* AGL-1 after verification by sequencing. The previously mentioned *bar* resistance and the resistance of *sur* to chlorimuron ethyl (10 μg/mL) were employed to screen the putative colonies of deletion mutants (DMs) and complementation mutants (CMs). Expected recombination events in DM, DDM (double-deletion mutant) and CM strains were examined through detection of the targeted DNA fragments using PCR (Figure S1E–H), and the transcriptional expression of each target gene in cDNA samples was examined through real-time quantitative PCR (qPCR) analysis (Figure S1I–L). All pairs of primers used in the vector construction and detection of *rad5*, *rad16A* and *rad16B* are listed in Table S1. The cDNA samples used in qPCR were derived from the 3-day-old SDAY cultures of targeted gene mutants and WT incubated at the optimal regime. The fungal β-actin gene was used as a reference. The relative transcript levels of *rad5*, *rad16A* and *rad1B* in the corresponding mutants versus WT were computed using a method of threshold cycle (2^−ΔΔCt^). The paired DM/CM strains of *rad5* and *rad16B*, three *rad14* DM strains (DM1–3), three *rad16A*/*B* DDM strains (DDM1–3), and the parental WT were used in all experiments consisting of three independent replicates.

### 2.6. Experiments for Lifecycle-Related Phenotypes

The experiments including three independent replicates were carried out to assess phenotypes associated with the life cycles of all DM/DDM and control (WT and CM) strains. The radial growth of each strain was initiated by spotting ~10^3^ conidia (in 1 μL of 10^6^ conidia/mL suspension) on the plates of 1/4 SDAY, CDA and amended CDAs, or on the plates of CDA alone (control) or supplemented with each of two oxidants, osmotic agents and cell wall stressors. All plates were incubated for 7 days at 25 °C and 12:12 (L/D), followed by measuring colony diameters as an index of radial growth under normal or stressful conditions. The relative growth inhibition (%) of each strain by each stress was assessed using the diameters of control and stressed CDA colonies.

To assess the conidiation capacity, conidial yields (no. conidia/cm^2^) were assessed from three SDAY cultures of each strain, which were incubated for 5, 7 and 9 days at 25 °C and 12:12 (L/D), respectively, after 100 μL of a 10^7^ conidia/mL suspension was spread per plate for initiation of each culture. The time (h) required for 50% germination (GT_50_) of conidia on GM plates at 25 °C was assessed as an index of conidial viability. Then, standardized bioassays were carried out to assess the conidial infectivity to *Galleria mellonella* larvae (last instar) through normal cuticle infection or cuticle-bypassing infection. The infection of each strain was initiated through the immersion of three groups of ~35 larvae for 10 s in 40 mL aliquots of conidial suspension (10^7^ conidia/mL) or injecting ~500 conidia (in 5 μL of 10^5^ conidia/mL suspension) into the hemocoel of each larva in each group. The mortality in each group was then monitored at 12 or 24 h intervals at 25 °C, followed by an estimation of median lethal time (LT_50_ in days) as an index of virulence in either infection mode through a modeling analysis of the time–mortality trend in each group.

### 2.7. Assays for the Indices of NER and Photorepair Activities

The UVB assays for conidial response were performed in a Bio-Sun^++^ UV chamber (Vilber Lourmat, Marne-la-Vallée, France), a device that generates UVB irradiations (weighted wavelength: 312 nm) of 0.1–1.6 J/cm^2^ within 0.75–10.2 min with an error control of ≤1 μJ/cm^2^ (10^−6^) per dose [62]. In brief, 100 μL of conidial suspension (10^7^ conidia/mL) was smeared evenly onto each of the GM plates. Then, after being dried in air with 10 min sterile ventilation to minimize the surface moisture, the plates, free of lids, were exposed to UVB irradiation at each of the 16 gradient UVB doses (0.03–0.8 J/cm^2^; *d*); three plates were irradiated for each strain at each dose. Then, covered with lids, the irradiated plates were incubated in full darkness for 12, 24 and 40 h at 25 °C to reactivate the UVB-impaired conidia through NER (dark reactivation). Alternatively, the irradiated plates were incubated under white light for 3 or 5 h at 25 °C and then in the dark for 9 and 21 h or 7 and 19 h to photoreactivate those impaired or inactivated conidia through photorepair at the respective regimes of L/D 3:9, 3:21, 5:7 and 5:19, leading to a total of 12 or 24 h incubation. After an 8 h incubation, percent germination was monitored at 2-h intervals until a maximum was present. In total, three fields of microscopic view per plate were observed for the counts of germinated and non-germinated conidia. The germination status was photographed at the end of each dark or L/D treatment. The maximal germination percentages of each strain from three plates not exposed to UVB were used as the controls. In each treatment after irradiation at each UVB dose, the ratio of maximal germination percentage of irradiated conidia over that of non-irradiated conidia was computed as the conidial survival index (*I*_s_). The *d*-*I*_s_ trend fit the modified equation *I*_s_ = 1/[1 + exp(*a* + *rd*)] [62] to solve parameters *a* and *r*. Based on the fitted equation, the median lethal UVB dose (LD_50_) to cause 50% viability loss of irradiated conidia was estimated at *I*_s_ = 0.5 (LD_50_ = −*a/r*). The LD_50_ estimates of irradiated conidia were considered as the indices of NER activities in the 12, 24 and 40 h dark reactivation and of photorepair activities in photoreactivation at the L/D regimes.

### 2.8. Data Analysis

The variations in all phenotypic parameters, including fitted GT_50_, LT_50_ and LD_50_, were revealed through a one-factor (strains) analysis of variance (ANOVA), followed by a multiple comparison of differences among the tested fungal strains through Tukey’s honestly significant difference (HSD) test.

## 3. Results

### 3.1. Recognition and Domain Architectures of Fungal Rad5 and Rad16 Homologs

The Rad5 orthologs and two Rad16 paralogs were found in the genomes of the surveyed entomopathogenic and non-entomopathogenic ascomycetes. Either Rad5 orthologs or Rad16A and Rad16B paralogs were clustered to the phylogenetic clades associated with fungal lineages (Figures S2 and S3), suggesting that they exist widely in ascomycetes. However, no homolog of the yeast Rad7 (NP_012586, 565 aa) as a core partner of Rad16 was found in the filamentous ascomycetes that harbor Rad16A and Rad16B. This implies that the two Rad16 paralogs could function distinctively in fungi other than the yeast.

**Figure 1:**
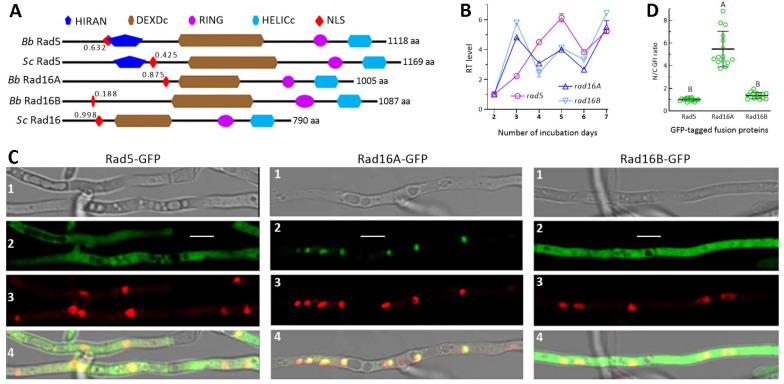
Recognition of Rad5, Rad16A and Rad16B in *B. bassiana*. (**A**) Comparison of domain architectures for Rad5 and Rad16 homologs of *B. bassiana* (*Bb*) and *S. cerevisiae* (*Sc*). Associated with each NLS motif is the maximal probability (decimal value) predicted. (**B**) Relative transcript (RT) levels for *rad5*, *rad16A* and *rad16B* expressed in the WT strain during 7-day incubation (versus day 2) on SDAY at 25 °C and 12:12 (L/D) photoperiod. (**C**) Laser scanning confocal microscopic images (scale: 5 μm) for subcellular localization of Rad5-GFP, Rad16A-GFP and Rad16B-GFP, which were expressed in the WT hyphae stained with DAPI. The bright, expressed (green), stained (red) and merged views of each microscopic field are shown in images 1, 2, 3 and 4, respectively. (**D**) N/C-GFI ratios of Rad5-GFP, Rad16A-GFP and Rad16B-GFP fusion proteins. Different uppercase letters denote a significant difference of *p* < 0.01 (Tukey’s test). Error bars: standard deviations (SDs) from three cDNA samples (**C**) or 15 hyphal cells (**D**) analyzed.

In *B. bassiana*, the Rad5 ortholog (EJP67286, 1118 aa) showed a sequence identity of 33.2% to the yeast query (total score 535, coverage 85%, e-value 5 × 10^−80^); the sequence identities of Rad16A and Rad16B to the yeast query were 55.4% (total score 749, coverage 88%, e-value zero) and 27.6% (total score 273, coverage 73%, e-value 7 × 10^−64^), respectively. Rad5 was revealed as having a HIRAN domain at the N-terminus, a DEXDc domain in the central region, and RING and HELICc domains at the C-terminus in the same way as the yeast query (Figure 1A). The mentioned domains except the N-terminal DEXDc were also found in the sequences of Rad16A, Rad16B and the yeast query. In addition, the NLS motifs of Rad5 or Rad16A versus the yeast query were predicted at maximal probability close to each other (0.632 versus 0.435 or 0.875 versus 0.998), contrasting with a low probability (0.188) associated with the NLS motif of Rad16B. These analyses reveal similar domain architectures for either the Rad5 or Rad16 homologs in *B. bassiana* and *S. cerevisiae*. The maximal probability associated with the NLS motif of Rad16A is much higher than that associated with the Rad16B counterpart, suggesting a subcellular difference of them.

### 3.2. Transcriptional and Posttranslational Features of Rad5, Rad16A and Rad16B

The genes *rad5*, *rad16A* and *rad16B* were expressed consistently in the WT strain over a 7-day period of incubation at 25 °C and 12:12 (L/D) photoperiod (Figure 1B). Relative to day 2, the upregulated pattern of *rad5* over the days of incubation was different from similar patterns of *rad16A* and *rad16B*. Among the GFP-tagged fusion proteins expressed in WT, Rad5-GFP and Rad16B-GFP accumulated in the cytoplasm and nuclei of the DAPI-stained hyphae; meanwhile, Rad16A-GFP accumulated exclusively in the nuclei (Figure 1C). The mean (±SD) N/C-GFI ratio (*n* = 15) was up to 5.47 (±1.58) for Rad16A-GFP (Figure 1D); this ratio was significantly greater than 1.01 (±0.14) for Rad5-GFP or 1.36 (±0.28) for Rad16B-GFP (*p* < 0.01, Tukey’s HSD). Both the images and the ratios indicate a localization of either Rad5 or Rad16B in the hyphal cytoplasm and nuclei and a nucleus-specific localization of Rad16A in *B. bassiana*.

**Figure 2:**
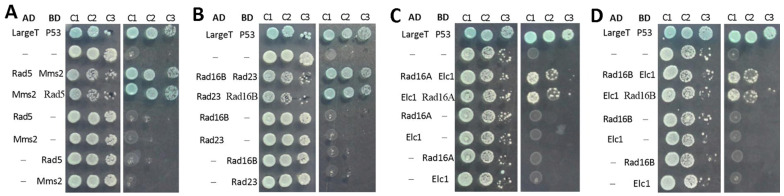
Y2H assays for protein–protein interactions. (**A**–**D**) Interactions of Rad5 (E3 ubiquitin ligase) with Mms2 (E2 ubiquitin-conjugating enzyme), Rad16B with Rad23, and Rad16A and Rad16B with the Elc1-like E3 ubiquitin ligase. The constructed diploids during 3 days of incubation at 30 °C grew as well as positive control on quadruple-dropout plates (right in each image) after inoculation with 5 × 10^2^ (C3), 5 × 10^3^ (C2) and 5 × 10^4^ (C1) cells.

In the Y2H assays, a strong interaction was detected between Rad5 and Mms2 (Figure 2A), a ubiquitin-conjugating E2 enzyme needed for error-free PPR in budding yeast [63], and between Rad16B and Rad23 (Figure 2B), a nucleocytoplasmic shuttling protein proven to interact with Phr2 and to have high photoreactivation activity in *B. bassiana* [52]. A protein resembling Elc1, an E3 ligase needed for the yeast GG-NER [64], also showed a positive interaction with either Rad16A (Figure 2C) or Rad16B (Figure 2D). However, Rad5, Rad16A and Rad16B were revealed as not interacting with one another or with any of the photolyases, photolyase regulators and other anti-UV proteins examined (Figure S4A–S). The anti-UV proteins included two paralogs (Clu3A/B) of Cul3 needed for the formation of a cullin-cored E3 ligase complex which facilitates the UV-dependent ubiquitination of Rad4 and proteins associated with the yeast chromatin [36,37]. These data demonstrate that Rad5, Rad16A and Rad16B could be functionally independent of each other in *B. bassiana*. The detected interactions of Rad5 with Mms2 and of either Rad16A or Rad16B with the Elc1-like protein were seemingly simplified in *B. bassiana* in comparison to those of their homologs detected previously in *S. cerevisiae* [36,37,41,42,43].

### 3.3. Negligible Roles of Rad5, Rad16A and Rad16B in Fungal Lifecycles

**Figure 3:**
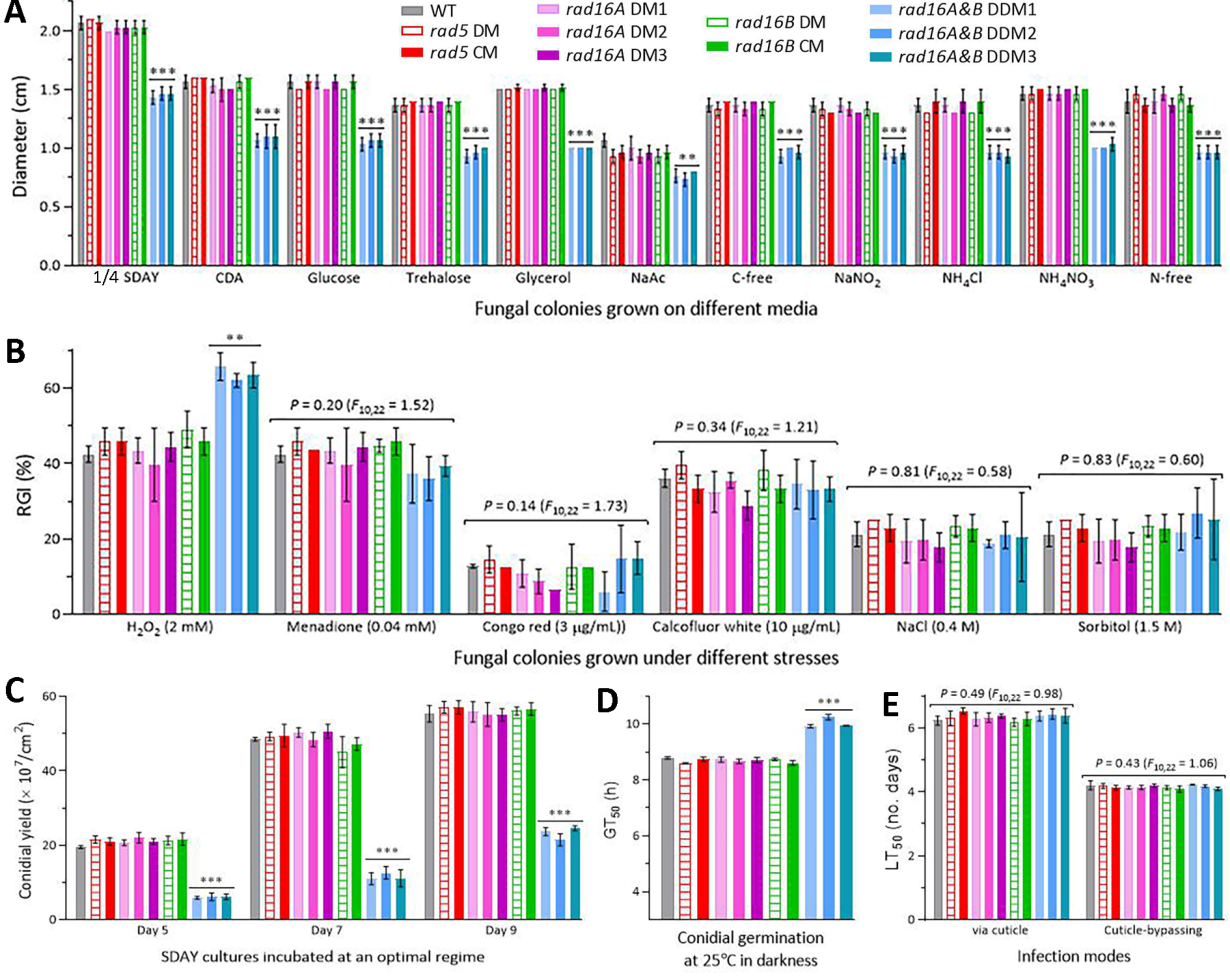
Roles of Rad5, Rad16A and Rad16B in *B. bassiana* lifecycle. (**A**) Diameters of fungal colonies (DM, deletion mutant; DDM, double-deletion mutant; CM, complementation mutant) incubated at the optimal regime of 25 °C and 12:12 (L/D) for 7 days on the plates of 1/4 SDAY, CDA and CDAs amended with different carbon or nitrogen sources. (**B**) Relative growth inhibition (RGI) of fungal colonies after 7-day incubation at 25 °C on CDA plates containing indicated concentrations of different chemical stressors. The colony growth was initiated with ~10^3^ conidia. (**C**) Conidial yields in the SDAY cultures incubated for 5, 7 and 9 days at the optimal regime after each culture was initiated by spreading 100 μL of conidial suspension (10^7^ conidia/mL). (**D**) Estimates of GT_50_ as an index of conidial viability. (**E**) Estimates of LT_50_ as a virulence index of fungal strains against *G. mellonella* larvae through normal cuticle infection or cuticle-bypassing infection (intrahemocoel injection). *p* < 0.01 ** or 0.001 *** in Tukey’s test. Error bars: SDs from three independent replicates.

The DM strains of *rad5*, *rad16A* and *rad16B* exhibited the same lifecycle-related phenotypes as the control strains, including radial growth on all tested media under normal culture conditions (Figure 3A); percent growth inhibition under oxidative, cell wall perturbing and osmotic stresses (Figure 3B); conidial yields from the SDAY cultures incubated for 5, 7 and 9 days at the optimal regime (Figure 3C); conidial viability denoted by GT_50_ (Figure 3D); and virulence denoted by LT_50_ via normal cuticle infection or cuticle-bypassing infection (Figure 3E). Compared to all DM and control strains, the three DDM strains of *rad16A* and *rad16B* showed significant defects (*p* < 0.01, Tukey’s HSD) in radial growth, tolerance to oxidative stress induced by H_2_O_2_, conidiation, and conidial viability. However, the DDM strains displayed null responses to the stresses induced by menadione and other chemical agents, and their virulence was unaffected in either infection mode. These data demonstrated the negligible roles of Rad5, Rad16A and Rad16B in the asexual and insect-pathogenic lifecycles of *B. bassiana*.

### 3.4. Low Activities of Rad5, Rad16A and Rad16B in Dark Reactivation

**Figure 4:**
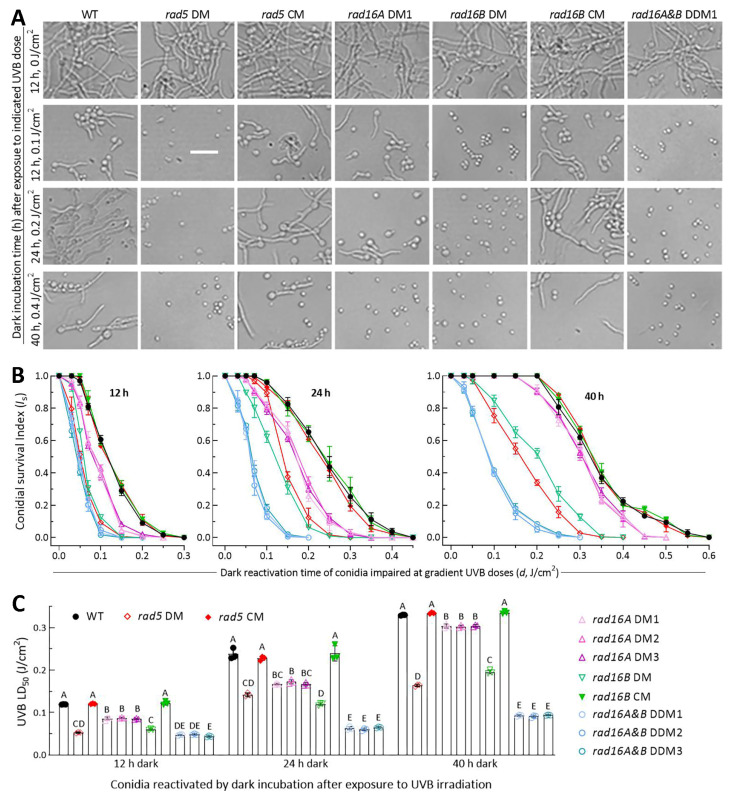
Comparative activities of Rad5, Rad16A and Rad16B in dark reactivation of *B. bassiana* conidia impaired by UVB to different degrees. (**A**) Microscopic images (scale: 20 μm) for germination status of UVB-impaired conidia after 12, 24 and 40 h of dark incubation at 25 °C. (**B**) Trends of conidial survival indices in the 12, 24 and 40 h dark treatments after exposure to gradient UVB doses. (**C**) LD_50_ values estimated as an index of NER activity from the survival trends fitted in different dark treatments. Different uppercase letters denote significant differences of *p* < 0.01 (Tukey’s HSD). Error bars: SDs from three independent replicates.

Almost all conidia produced via the DM, DDM and control strains germinated after 12 h dark incubation at the optimal 25 °C (Figure 4A). The control strains’ conidia were largely reactivated (germinated) at the end of 12 h of dark incubation after exposure to a UVB dose of 0.1 J/cm^2^ or of 24 h of dark incubation after exposure to 0.2 J/cm^2^; however, the conidia were less reactivated at the end of 40 h of dark incubation after exposure to 0.4 J/cm^2^. The dark-reactivated conidia were observable for *rad16A* DM, but hardly observed for the other DM and DDM strains, after exposure to 0.1 or 0.2 J/cm^2^. For all DM and DDM strains, the conidia impaired at 0.4 J/cm^2^ were not reactivated at all through 40 h of dark incubation.

The dark reactivation rates of the impaired conidia varied with increases in the UVB dose and dark incubation time and showed declining trends that were markedly different among the tested strains (Figure 4B–D). For all tested strains, the UVB dose-dependent survival trends of those differentially impaired conidia in the 12, 24 and 40 h dark treatments fit the mentioned equation well (r^2^ ≥ 0.97, *p* < 0.001 for fitness *F* tests), leading to an LD_50_ estimate of each strain as an index of NER activity in each of the three dark treatments. The LD_50_ values (*n* = 9) of three control strains in the 12, 24 and 40 h dark treatments averaged 0.121 (±0.003), 0.235 (±0.013) and 0.333 (±0.003) J/cm^2^ (Figure 4E), respectively. Compared to these means, the LD_50_ estimates in the three dark treatments were reduced significantly (*p* < 0.01 in Tukey’s test) by 56%, 40% and 51% in *rad5* DM; 29%, 28% and 9% in *rad16A* DM1–3; 50%, 49% and 41% in *rad16B* DM; and 61%, 73% and 72% in DDM1–3, respectively.

The LD_50_ values indicated greater NER activities of Rad5 and Rad16B than of Rad16A and additive NER activities of Rad16A and Rad16B in each of the three dark treatments. Notably, the LD_50_ value of each DM/DDM or control strain was significantly increased with an increase in the dark incubation time. This implied that the NER activity of each target protein was limited to reactivation of low UVB dose-impaired conidia via 12 h dark incubation mimicking natural night but was unable to reactivate conidia severely impaired at higher UVB doses unless the night/dark time was doubled or further prolonged.

### 3.5. High Activities of Rad5, Rad16A and Rad16B in Photoreactivation

**Figure 5:**
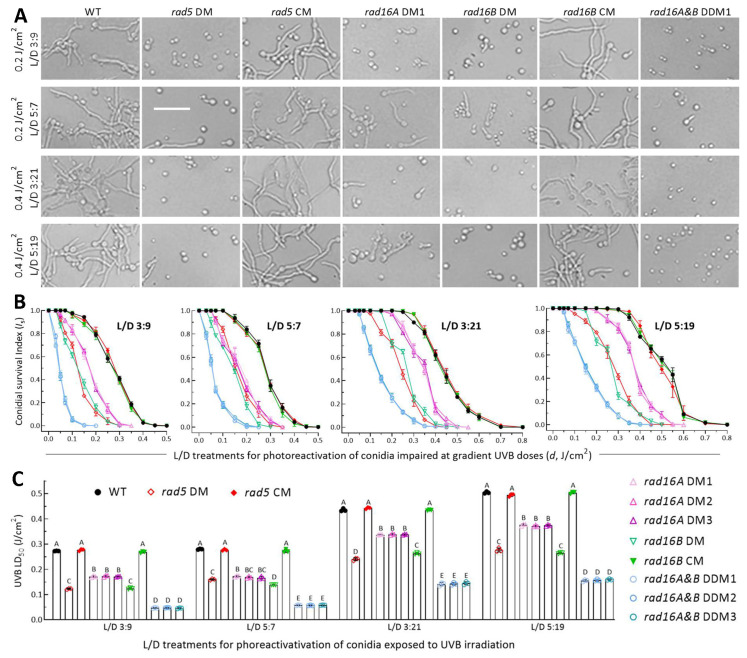
Comparative activities of Rad5, Rad16A and Rad16B in photoreactivation of *B. bassiana* conidia irradiated at gradient UVB doses. (**A**) Microscopic images (scale: 20 μm) for germination status of UVB-impaired conidia incubated at 25 °C for a total of 12 or 24 h in L/D 3:9, 5:7, 3:21 and 5:19 post-irradiation at 0.2 or 0.4 J/cm^2^, respectively. (**B**) Trends of conidial survival indices photoreactivated in the L/D treatments after conidia were irradiated at gradient UVB doses. (**C**) LD_50_ values estimated as an index of photorepair activity from the survival trends fitted in different L/D treatments. Different uppercase letters denote significant differences of *p* < 0.01 (Tukey’s HSD). Error bars: SDs from three independent replicates.

Most of the control strains’ conidia were photoreactivated under a regime of L/D 3:9 or 5:7 after exposure to a UVB dose of 0.2 J/cm^2^ or at L/D 3:21 or 5:19 after exposure to a lethal dose of 0.4 J/cm^2^ (Figure 5A). In the L/D treatments, the photoreactivation rates of irradiated conidia declined much more slowly with increasing UVB doses in the control strains than in the DM strains or three DDM strains, which showed similar trends of a sharp decrease (Figure 5B–E). The photoreactivation rates under the UVB doses of ≤0.5 or ≤0.8 J/cm^2^ tested in the L/D treatments fit the equation well (r^2^ ≥ 0.98, *p* < 0.001 for fitness *F* tests). The LD_50_ values (*n* = 9) of the control strains estimated from the fitted equations averaged 0.272 (±0.005) J/cm^2^ at L/D 3:9, 0.277 (±0.005) J/cm^2^ at L/D 5:7, 0.438 (±0.005) J/cm^2^ at L/D 3:21 and 0.501 (±0.006) J/cm^2^ at L/D 5:19 (Figure 5F). The four L/D treatments led to mean LD_50_ reductions by 55%, 42%, 45% and 45% in the absence of *rad5*; 37%, 39%, 23% and 25% in the absence of *rad16A*; 54%, 50%, 44% and 47% in the absence of *rad16B*; and 83%, 79%, 67% and 69% in the three DDM strains of *rad16A* and *rad16B*, respectively, in comparison to those from the control strains. All the reductions were significant at *p* < 0.01 (Tukey’s HSD).

The different LD_50_ estimates of the DM/DDM and control strains in the L/D treatments indicated greater activities of Rad5 and Rad16B than of Rad16A and additive activities of Rad16A and Rad16B in the photoreactivation of UVB-impaired or inactivated conidia. Notably, the LD_50_ value of each DM, DDM or control strain at L/D 3:9 or 5:7 was twice or twice more of that achieved with 12 h of dark incubation. This indicated the more significant activity of Rad5, Rad16A or Rad16B in photorepair-dependent photoreactivation than in NER-dependent dark reactivation for the feasible recovery of *B. bassiana* from solar UV damage on the Earth’s surface. In other words, their anti-UV roles in *B. bassiana* principally depend on photoreactivation in vivo.

## 4. Discussion

In *B. bassiana*, none of the *rad5*, *rad16A* and *rad16B* DM strains versus the control strains were affected in main phenotypes related to the fungal asexual and insect-pathogenic lifecycles. Intriguingly, some of the examined phenotypes were similarly defective in the DDM strains of *rad16A* and *rad16B*, including slower growth, decreased conidiation compacity, and increased sensitivity to H_2_O_2_-induced stress. These phenotypic defects hint at that the coexistence of Rad16A and Rad16 is biologically important for the insect pathogen. Despite having no impact on the fungal lifecycle-related phenotypes, singular disruptions of *rad5*, *rad16A* and *rad16B* resulted in differentially reduced anti-UVB activities in either the dark reactivation or photoreactivation of conidia impaired with UVB to different degrees; the reduction was enlarged via the double disruption of *rad16A* and *rad16B*, as discussed below.

The interaction of Rad5 with Mms2 rather than Rad18 in *B. bassiana* is distinct from the documented interaction of Rad5 with Rad18 to form a protein complex comprising Rad5, Rad6-Rad18 and Ubc13-Mms2 needed for the bypass of DNA lesions through error-free PRR in the yeast [41,42,43]. Rad16A and Rad16B not interacting with each other lack the core partner Rad7 that interacts with the Rad16 ortholog in the yeast to form the cullin-based E3 ligase complex required for GG-NER [36,37]. In particular, nucleocytoplasmic shuttling Rad16B was found interacting with Rad23, another nucleocytoplasmic shuttling protein that has been proven to interact with Phr2 and have high photoreactivation activity in *B. bassiana* [52]. However, either Rad16A or Rad16B was revealed to not interact with Rad4A, which interacts with Rad23 and shows photoreactivation activity in *B. bassiana* [57]. This is different from the yeast cullin-based E3 complex comprising Rad4 and Rad7-Rad16 [36,37]. In *B. bassiana*, either nucleocytoplasmic Rad16B or nucleus-specific Rad16A were shown to interact with the Elc1-like E3 ligase but not with either paralog of Cul3, which interacts with Elc1 and Rad7-Rad16 to form the yeast E3 complex. Apparently, the protein–protein interactions comprising Rad16 in the yeast are largely simplified for those of Rad16A or Rad16B that were detected in this study. The simplified interactions led to differential activities of Rad5, Rad16A and Rad16B in the dark reactivation and photoreactivation of UVB-impaired *B. bassiana*, respectively.

The UVB LD_50_ values of all tested strains in three dark and four L/D treatments confirmed much higher activities of Rad5, Rad16A and Rad16B in photoreactivation than in dark reactivation to aid recovery of solar UV damage. Rad5 and Rad16B showed greater anti-UVB effects than did Rad16A. Interestingly, either the dark reactivation or photoreactivation activities of the two Rad16 paralogs were additive in each dark or L/D treatment, suggesting an importance of their coexistence for the fungal anti-UV effect. However, their activities in 12 h of dark reactivation mimicking a natural night were insufficient to recover severe UVB damage unless the dark period was prolonged to 24 h or longer, which is unavailable on the Earth’s surface. In contrast, their photoreactivation activities under the regime of L/D 3:9 or 5:7 were able to recover sublethal or lethal UVB dose-impaired conidia. According to the differences in photoreactivation rates between the DM and control strains in the L/D treatments, Rad5 and Rad16B exhibited photoreactivation activities close to those of Rad1, Rad2, Rad10, Rad14 and Rad23 in *B. bassiana* [54,57,60] and those of Rad1, Rad2, Rad4A, Rad10, Rad14 and Rad26 in *M. robertsii* [56,58,59]. Rad16A was inferior to Rad5 or Rad16B in the photoreactivation in vivo.

Finally, the solar UVB dose accumulated daily on the sunny days of summer is several times above conidial tolerance [1], within which UVB damage is recoverable via photoreactivation in insect-pathogenic fungi [50,52,53,54,55,56,57,58,59,60]. More than 90% of the solar UVB dose accumulates prior to 3:00 p.m., contrasting with an accumulation of ~0.2 J/cm^2^ after 3:00 p.m. [1]. The inference obtained from the photoreactivation activities of Rad5, Rad16A and Rad16B characterized in this study and of other anti-UV proteins shown in recent studies is that solar UVB damage accumulated after 3:00 p.m. is easily recovered via light exposure over a period of pre-evening in the daytime. Therefore, the field application of fungal insecticides based on formulated conidia should not be scheduled over a period from early morning to 3:00 p.m. to avoid increasing UVB damage and should be scheduled after 3:00 p.m. because conidia impaired via a solar UVB dose of ~0.2 J/cm^2^ can be photoreactivated with several hours of pre-evening daylight and ~10 h of subsequent nighttime. A period from late afternoon to the early morning of the following day is long enough for the germination of applied conidia to initiate hyphal invasion into the insect body. For this reason, two fungal insecticides sprayed in the late afternoon have proved more efficacious for the control of rice planthoppers and leaf rollers than the counterparts sprayed in the morning for increased exposure to solar UV on the sunny days of summer [65,66].

It is conclusive that the anti-UV effects of Rad5, Rad16A and Rad16B in *B. bassiana* depend primarily on their activities in photorepair-dependent photoreactivation. Their activities in dark reactivation are operative but cannot recover severe UVB damage as do their photoreactivation activities unless the night/dark period exceeds 24 h, which is not feasible in the field. These findings expand further on the molecular basis for the recovery of insecticidal fungal cells from solar UV damage through photoreactivation and unveil a scenario that is distinct from the anti-UV effects for the yeast Rad5 and Rad7-Rad16 to depend on error-free PRR and GG-NER, respectively [18]. This expanded basis is exploitable for the rational application of mycoinsecticides to minimize or avoid direct exposure to solar UV irradiation on the sunny days of summer.

## Figures and Tables

**Table 1:** A list of microbial strains and media used in the present study.

Strain (Abbreviated) Name	Medium (Abbreviated) Name	Purpose
*B. bassiana* ARSEF2860 wild-type strain (WT)	Sabouraud dextrose agar (*w*/*v*: 4% glucose, 1% peptone and 1.5% agar) plus 1% yeast extract (SDAY), 1/4 SDAY (amended with 1/4 nutrition strength of SDAY)	Gene manipulation, growth and conidiation
Targeted gene deletion mutants (DMs)	Czapek-Dox agar (CDA; *w*/*v*: 3% sucrose, 0.3% NaNO_3_, 0.1% K_2_HPO_4_, 0.05% KCl, 0.05% MgSO_4_ and 0.001% FeSO_4_ plus 1.5% agar), CDAs amended with different carbon or nitrogen sources	Radial growth, stress response
Targeted gene complementation mutants (CMs)	Germination medium (GM; *w*/*v*: 2% sucrose, 0.5% peptone and 1.5% agar)	Germination after UVB irradiation
*S. cerevisiae* Y187 and Y2HGold	YPD (*w*/*v*: 1% yeast extract, 2% peptone, 2% glucose plus 0.04% adenine hemisulfate salt)	Yeast two-hybrid (Y2H) assays
*Escherichia coli* DH5α	Luria-Bertani medium containing ampicillin (100 mg/mL) or kanamycin (50 mg/mL)	Vector propagation
*Agrobacterium tumefaciens* AGL-1	YEB (*w*/*v*: 0.5% sucrose, 0.1% yeast extract, 1% peptone, 0.05% MgSO_4_, 1.5% agar)	Fungal transformation

## Data Availability

All experimental data are included in this paper and Supplementary Material.

## Supplementary Materials

The following supporting information can be downloaded at https://www.mdpi.com/article/10.3390/jof10060420/s1, Table S1: Paired primers used for manipulation and detection of target genes in *B. bassiana*; Figure S1: Generation and identification of *rad5*, *rad16A* and *rad16B* mutants in *B. bassiana*; Figure S2: Phylogenetic relationships of Rad5 orthologs found in selected ascomycetes; Figure S3: Phylogenetic relationships of Rad16 homologs found in selected ascomycetes; Figure S4: Y2H assays for negative protein-protein interactions in *B. bassiana*.
