# Supplementary material for: Photoreactivation Activities of Rad5, Rad16A and Rad16B Help Beauveria bassiana to Recover from Solar Ultraviolet Damage
Source: J Fungi (Basel). 2024 Jun 13;10(6):420. doi: 10.3390/jof10060420 (PMC11205155; doi:10.3390/jof10060420)
Supplement: Supplementary file 1 [file jof-10-00420-s001.zip › jof-2977134-supplementary.pdf]

Supplementary Material

Photoreactivation Activities of Rad5, Rad16A and Rad16B Help *Beauveria bassiana* to recover from Solar Ultraviolet Damage

Xin-Cheng Luo, Lei Yu, Si-Yuan Xu, Sheng-Hua Ying, and Ming-Guang Feng*

Institute of Microbiology, College of Life Sciences, Zhejiang University, Hangzhou, Zhejiang, 310058, China

(*Address correspondence to Ming-Guang Feng, mgfeng@zju.edu.cn)

Table S1. Paired primers used for manipulation and detection of target genes in *B. bassiana*.

Primers	Paired sequences (5′–3′) [a]	Purpose [b]
cRad5-F/R	CAATCACAAACACCTTCAAAATGGACTTTGCCAACTCACA / TCCTCGCCCTTGCTCACCATGCTAAGCAGTTCTTTCATGT	Cloning *rad5* cDNA (3357 bp) for fusion to *GFP*
cRad16B-F/R	CAATCACAAACACCTTCAAAATGCAGTCCTGGAACCCAGC/ TCCTCGCCCTTGCTCACCATCTTGAACAGGCTGCGGATAT	Cloning *rad16B* cDNA (3264 bp) for fusion to *GFP*
cRad16AF/R	C AATCACAAACACCTTCAAAATGCGTCGTGGACGGTCTTC / TCCTCGCCCTTGCTCACCATTTTGCGAAGCTTTGCCAGCG	Cloning *rad16A* cDNA (3018 bp) for fusion to *GFP*
adRad5-F/R	GCCATGGAGGCCAGT**GAATTC**ATGGACTTTGCCAACTCACA / CAGCTCGAGCTCGAT**GGATCC**GCTAAGCAGTTCTTTCATGT	Cloning *rad5* cDNA (3357 bp) for ligation to AD
bdRad5-F/R	ATGGCCATGGAGGCC**GAATTC**ATGGACTTTGCCAACTCACA / CGCTGCAGGTCGAC**GGATCC**GCTAAGCAGTTCTTTCATGT	Cloning *rad5* cDNA (3357 bp) for ligation to BD
adRad16A-F/R	GCCATGGAGGCCAGT**GAATTC**ATGCGTCGTGGACGGTCTTC / CAGCTCGAGCTCGAT**GGATCC**ATTACCACGGAACAAAAACT	Cloning *rad16A* cDNA (3018 bp) for ligation to AD
bdRad16A-F/R	ATGGCCATGGAGGCC**GAATTC**ATGCGTCGTGGACGGTCTTC / CGCTGCAGGTCGAC**GGATCC**ATTACCACGGAACAAAAACT	Cloning *rad16A* cDNA (3018 bp) for ligation to BD
adRad16B-F/R	GCCATGGAGGCCAGT**GAATTC**ATGCAGTCCTGGAACCCAGC / CAGCTCGAGCTCGAT**GGATCC**CTTGAACAGGCTGCGGATAT	Cloning *rad16B* cDNA (3264 bp) for ligation to AD
bdRad16B-F/R	ATGGCCATGGAGGCC**GAATTC**ATGCAGTCCTGGAACCCAGC / CGCTGCAGGTCGAC**GGATCC**CTTGAACAGGCTGCGGATAT	Cloning *rad16B* cDNA (3264 bp) for ligation to BD
adELC1-F/R	GCCATGGAGGCCAGT**GAATTC**ATGGCGACGACTCAGACTCC / CAGCTCGAGCTCGAT**GGATCC**GTTTGTCATGATAGCTTGGTCCAA	Cloning *ELC1* cDNA (333 bp) for ligation to AD
bdELC1-F/R	ATGGCCATGGAGGCC**GAATTC**ATGGCGACGACTCAGACTCC / CGCTGCAGGTCGAC**GGATCC**GTTTGTCATGATAGCTTGGTCCAA	Cloning *ELC1* cDNA (333 bp) for ligation to BD
adCul1-F/R	GCCATGGAGGCCAGT**GAATTC**ATGATCTCTGGGAGAGGAAG / CAGCTCGAGCTCGAT**GGATCC**CGCCACATAGCGGTACAC	Cloning *cul3A* cDNA (2520 bp) for ligation to AD
bdCul1-F/R	ATGGCCATGGAGGCC**GAATTC**ATGATCTCTGGGAGAGGAAG / CGCTGCAGGTCGAC**GGATCC**CGCCACATAGCGGTACAC	Cloning *cul3A* cDNA (2520 bp) for ligation to BD
adCul2-F/R	GCCATGGAGGCCAGT**GAATTC**ATGCACTCCCTCGCGGATCA / CAGCTCGAGCTCGAT**GGATCC**CGCCAAGTACGTGTACGCGT	Cloning *cul3B* cDNA (2493 bp) for ligation to AD
bdCul2-F/R	ATGGCCATGGAGGCC**GAATTC**ATGCACTCCCTCGCGGATCA / CGCTGCAGGTCGAC**GGATCC**CGCCAAGTACGTGTACGCGT	Cloning *cul3B* cDNA (2493 bp) for ligation to BD
adMMS2-F/R	GCCATGGAGGCCAGT**GAATTC**ATGGCCAAGGTTCCTCGTAA / CAGCTCGAGCTCGAT**GGATCC**TGAGTAGGTAGAACCCT	Cloning *mms2* cDNA (420 bp) for ligation to AD
bdMMS2-F/R	ATGGCCATGGAGGCC**GAATTC**ATGGCCAAGGTTCCTCGTAA / CGCTGCAGGTCGAC**GGATCC**TGAGTAGGTAGAACCCT	Cloning *mms2* cDNA (420 bp) for ligation to BD
adRad1-F/R	GCCATGGAGGCCAGT**GAATTC**ATGTCGACAAACAATGCGCC / CAGCTCGAGCTCGAT**GGATCC**GTAATCTTCTTCGTCCATGA	Cloning *rad1* cDNA (2820 bp) for ligation to AD
bdRad1-F/R	ATGGCCATGGAGGCC**GAATTC**ATGTCGACAAACAATGCGCC / CGCTGCAGGTCGAC**GGATCC**GTAATCTTCTTCGTCCATGA	Cloning *rad1* cDNA (2820 bp) for ligation to BD
adRad2 -F/R	GCCATGGAGGCCAGT**GAATTC**ATGGGCGTGAACGGTCTTTG / CAGCTCGAGCTCGAT**GGATCC**CTGCTTCCGCCGTTTATTGC	Cloning *rad2* cDNA (3462 bp) for ligation to AD
bdRad2 -F/R	ATGGCCATGGAGGCC**GAATTC**ATGGGCGTGAACGGTCTTTG / CGCTGCAGGTCGAC**GGATCC**CTGCTTCCGCCGTTTATTGC	Cloning *rad2* cDNA (3462 bp) for ligation to BD
adRad6 -F/R	GCCATGGAGGCCAGT**GAATTC**ATGTCAACGGCCGCCAGACG / CAGCTCGAGCTCGAT**GGATCC**GTCCTCCCAGCTCTTCTCTA	Cloning *rad6* cDNA (456 bp) for ligation to AD
bdRad6 -F/R	ATGGCCATGGAGGCC**GAATTC**ATGTCAACGGCCGCCAGACG / CGCTGCAGGTCGAC**GGATCC**GTCCTCCCAGCTCTTCTCTA	Cloning *rad6* cDNA (456 bp) for ligation to BD
adRad18 -F/R	GCCATGGAGGCCAGT**GAATTC**ATGCCTGTTGACGATGTTGCGGATT / CAGCTCGAGCTCGAT**GGATCC**TAAATACTGCTCTTGCCTGATTCCG	Cloning *rad18* cDNA (1275bp) for ligation to AD
bdRad18 -F/R	ATGGCCATGGAGGCC**GAATTC**ATGCCTGTTGACGATGTTGCGGATT / CGCTGCAGGTCGAC**GGATCC**TAAATACTGCTCTTGCCTGATTCCG	Cloning *rad18* cDNA (1275 bp) for ligation to BD
adRad10-F/R	GCCATGGAGGCCAGT**GAATTC**ATGGCTGACGAATACGGCGC / CAGCTCGAGCTCGAT**GGATCC**TTTGCGAAGCTTTGCCAGCG	Cloning *rad10* cDNA (1176 bp) for ligation to AD
bdRad10-F/R	ATGGCCATGGAGGCC**GAATTC**ATGGCTGACGAATACGGCGC / CGCTGCAGGTCGAC**GGATCC**TTTGCGAAGCTTTGCCAGCG	Cloning *rad10* cDNA (1176 bp) for ligation to BD
adRad14 -F/R	GCCATGGAGGCCAGT**GAATTC**ATGGAGCGCCCAAAGACGCC / CAGCTCGAGCTCGAT**GGATCC**CAGCTCCAGCTCCTCCACTT	Cloning *rad14* cDNA (1149 bp) for ligation to AD
bdRad14 -F/R	ATGGCCATGGAGGCC**GAATTC**ATGGAGCGCCCAAAGACGCC / CGCTGCAGGTCGAC**GGATCC**CAGCTCCAGCTCCTCCACTT	Cloning *rad14* cDNA (1149 bp) for ligation to BD
adRad23 -F/R	GCCATGGAGGCCAGT**GAATTC**ATGAAGGTCACCTTCAGAGA / CAGCTCGAGCTCGAT**GGATCC**TTGGCTCGCAGGCGGCTGCT	Cloning *rad23* cDNA (1191 bp) for ligation to AD
bdRad23 -F/R	ATGGCCATGGAGGCC**GAATTC**ATGAAGGTCACCTTCAGAGA / CGCTGCAGGTCGAC**GGATCC**TTGGCTCGCAGGCGGCTGCT	Cloning *rad23* cDNA (1191 bp) for ligation to BD

Table S1 (continued)

Primers	Paired sequences (5′–3′) [a]	Purpose [b]
adWC1-F/R	GCCATGGAGGCCAGT**GAATTC**ATGGAAGGATACTACCCTCC / CAGCTCGAGCT CGAT**GGATCC**AGGTAAGCTCGTTTCACGCT	Cloning *wc1* cDNA (2889 bp) for ligation to AD
adWC2-F/R	GCCATGGAGGCCAGT**GAATTC**ATGTCCCAGGGACACGCGCC / CAGCTCGAGC TCGAT**GGATCC**GCTTCCGGCACCAAACTCTG	Cloning *wc2* cDNA (1497 bp) for ligation to AD
adPhr1-F/R	GCCATGGAGGCCAGT**GAATTC**ATGGCGCCTCGTGCAACGAA / CAGCTCGAGC TCGAT**GGATCC** CGCCACAGCCGCCTTGTACG	Cloning *phr1* cDNA (1761 bp) for ligation to AD
bdPhr1-F/R	ATGGCCATGGAGGCC**GAATTC**ATGGCGCCTCGTGCAACGAA / CGCTGCAGGT CGAC**GGATCC**CGCCACAGCCGCCTTGTACG	Cloning *phr1* cDNA (1761 bp) for ligation to BD
adPhr2-F/R	GCCATGGAGGCCAGT**GAATTC**ATGACAAAGCCCAGAGTCAT / CAGCTCGAGCT CGAT**GGATCC**CGTTTTCTGCTTCTTCGCTG	Cloning *phr2* cDNA (1869 bp) for ligation to AD
bdPhr2-F/R	ATGGCCATGGAGGCC**GAATTC**ATGACAAAGCCCAGAGTCAT / CGCTGCAGGT CGAC**GGATCC**CGTTTTCTGCTTCTTCGCTG	Cloning *phr2* cDNA (1869 bp) for ligation to BD
bdWC1-F/R	ATGGCCATGGAGGCC**GAATTC**ATGGAAGGATACTACCCTCC / CGCTGCAGGTC GAC**GGATCC**AGGTAAGCTCGTTTCACGCT	Cloning *wc1* cDNA (2889 bp) for ligation to BD
bdWC2-F/R	ATGGCCATGGAGGCC**GAATTC**ATGTCCCAGGGACACGCGCC / CGCTGCAGGT CGAC**GGATCC**GCTTCCGGCACCAAACTCTG	Cloning *wc2* cDNA (1497 bp) for ligation to BD
upRad5-F/R	ACGAGCTGTACAAGTAA**CCCGGG**TGAGGCGTGAAATCCAGAA / TGGCTGCAG GTCGAC**GGATCC**CGAGCCGACCAATCTCCATAC	Cloning *rad5* 5'-end (1471bp) for disruption
dnRad5-F/R	GACCCATGGCTCGAG**TCTAGA**ACCAGTTTACCGACAAGCCA / GGTGGTGGTG GCTAGC**GTTAAC**GGACAAGAAAACCTCCATCAC	Cloning *rad5* 3'-end (1402 bp) for disruption
upRad16A-F/R	ACGAGCTGTACAAGTAA**CCCGGG**ACAAGTGAGAAAAGGGAACGTC / CGGTAC CAAGCTTGG**CTGCAG**AGTAGCAGAACCCGCAGAAG	Cloning *rad16A* 5'-end (1443 bp) for disruption
dnRad16A-F/R	GACCCATGGCTCGAG**TCTAGA**GTGGAGTGTTTCCTCGTTTCA / GGTGGTGGTG GCTAGC**GTTAAC**CAACACGGCAAGACAAGAATC	Cloning *rad16A* 3'-end (1409 bp) for disruption
upRad16B-F/R	AGCTGTACAAGTAA**CCCGGG**GCTGGCTGTGATAGGTGGAGTA / TGGCTGCAG GTCGAC**GGATCC**GTTTGTAACGGTGGTGAGATTG	Cloning *rad16B* 5'-end (1509 bp) for disruption
dnRad16B-F/R	GACCCATGGCTCGAG**TCTAGA**CATAACACGAAGCCCAAAAC / GGTGGTGGTG GCTAGC**GTTAAC**TGCTGGCAGGCAAGTATCT	Cloning *rad16B* 3'-end (1551 bp) for disruption
flRad5-F/R	ATCCGTCGACCTGCAGCC**AAGCTT**TGAGGCGTGAAATCCAGAAT/ ACACTAGTCAGA TCTTC**TCTAGA**AAGCCCTCGTCAAATACTGC	Cloning full-length *rad5* (4853 bp) for complementation
flRad16B-F/R	ATCCGTCGACCTGCAGCC**AAGCTT** TGTGACTGTTTCGCCTCTTC / ACACTAGTC AGATCT TC**TCTAGA**GTTTTGGGCTTCGTGTTATG	Cloning full-length *rad16B* (4515 bp) for complementation
upRad16A2-F/R	ACAGTACACGAGGACT**TCTAGA**ACAAGTGAGAAAAGGGAACGTC / CTGCCCC TGAGAG**GAATTC**AGTAGCAGAACCCGCAGAAG	Cloning *rad16A* 5'-end (1443 bp) for double disruption
dnRad16A2-F/R	CCGTCTCTCCGCATGC**ACTAGT**GTGGAGTGTTTCCTCGTTTCA / GGTGGTGGTG GCTAGC**GTTAAC**CAACACGGCAAGACAAGAATC	Cloning *rad16A* 3'-end (1409 bp) for double disruption
pRad5-F/R	GTTGGTGAGAAGGTGACGAAG/ TCCACCAAGGGTCCATCAT	PCR detecting *rad5*
pRad16A-F/R	GTGCCTACACCGCTATCAACA/ CTTTGTCATCGCCATTCACC	PCR detecting *rad16A*
pRad16B-F/R	TGTGACTGTTTCGCCTCTTC / GTTTTGGGCTTCGTGTTATG	PCR detecting *rad16B*
qRad5-F/R	CCCCGAAAACCAAGGCAAAG/AACGCTTCCGTCAAACTTGC	qPCR detecting *rad5*
qRad16A-F/R	TCAACTGACGAACCCGACTG/TCGTGCACCAAGAGCTCAAT	qPCR detecting *rad16A*
qRad16B-F/R	CAAGCGCCATCATTACCACG/GGTGGTGGCGACTTTTGTTC	qPCR detecting *rad16B*
qActin-F/R	GGCAACATTGTCATGTCTGG / TTTGCTGGAAGGTGGATAGG	qPCR detecting β-actin gene

[a] Underlined regions denote DNA fragments to exchange for the corresponding fragments of constructed vectors at the sites (in bold) of restriction enzymes for ligation to AD or BD (*Eco*RI/*Bam*HI), homologous recombination of 5′ and 3′ DNA fragments of *rad5, rad16A* or *rad16B* (*Xma*II/*Bam*HI and *Xba*I/*Hpa*I) in the WT background or 5′ and 3′ DNA fragments of *rad16A* in the null mutant of *rad16B* (E*co*RI/*Xba*I and *Spe*I/*Hpa*I), or complementation of *rad5* or *rad16B* (HindIII/XbaI).

[b] Tag loci for cloned genes: *rad5* (BBA_03842), *rad16A* (BBA_07794), *rad16B* (BBA_01511), *phr1* (BBA_01664), *phr2* (BBA_01034), *wc1* (BBA_10271), *wc2* (BBA_01403), *rad1* (BBA_07749), *rad10* (BBA_03417), *rad2* (BBA_00274), *rad6* (BBA_01469), *rad18* (BBA_03508), *rad14* (BBA_03454), *rad4A* (BBA_02814), *rad23* (BBA_01030), *mms2* (BBA_08836), *elc1* (BBA_03154), *cul3A* (BBA01191), *cul3B* (BBA_02238).

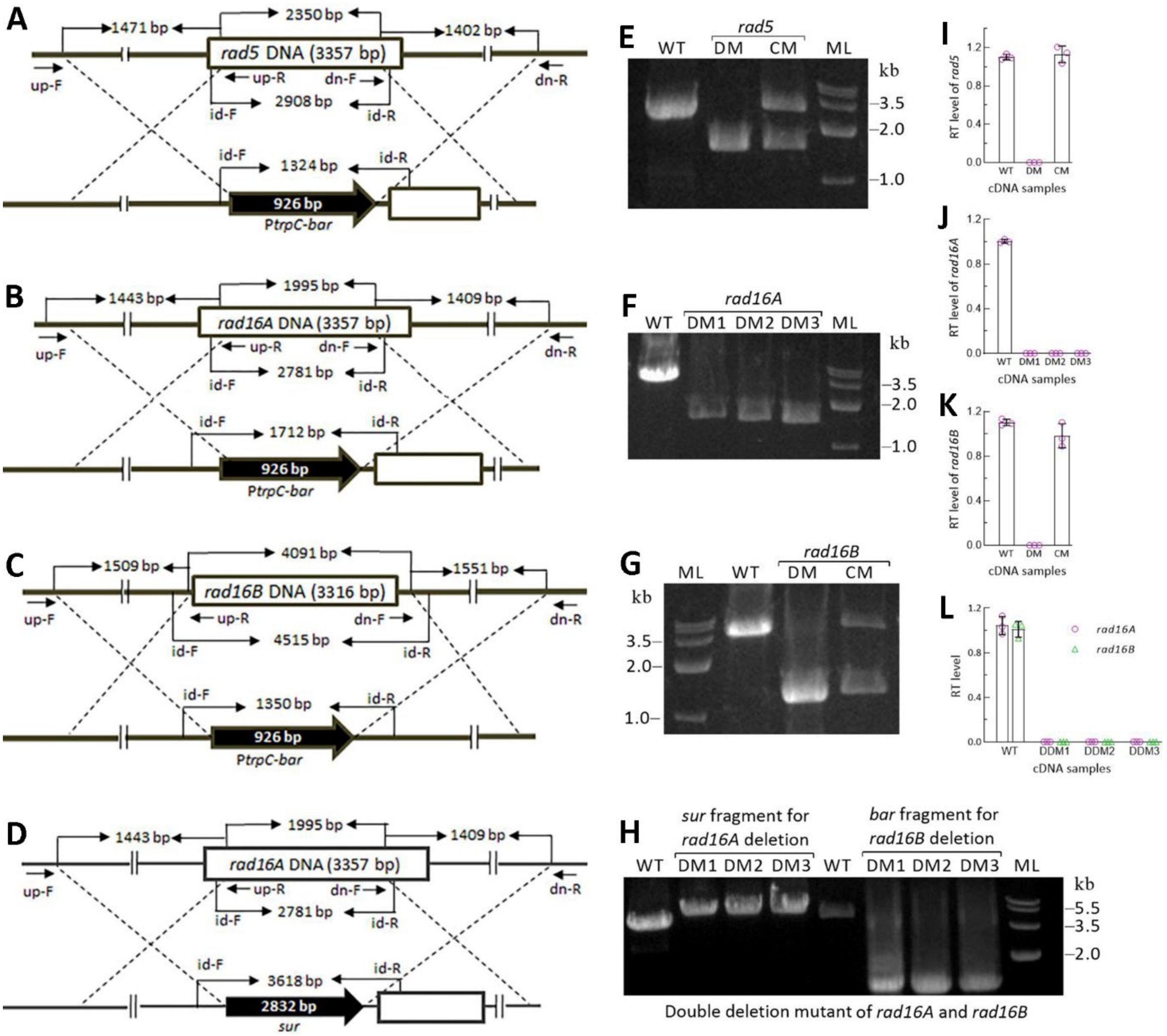

Figure S1. Generation and identification of *rad5, rad16A* and *rad16B* mutants in *B. bassiana*. (**A–C**) Schematic diagrams for the strategies of singular *rad5*, *rad16A* and *rad16B* deletions in the WT background. (**D**) Schematic diagram for the strategy of *rad16A* deletion in the null mutant of *rad16B*. (**E–H**) The *rad5, rad16A* and *rad16B* mutants identified via PCR analysis with paired primers (Table S1), respectively. The detected DNA fragments indicate a success in deleting the partial or full-length coding and partial flanking regions of each target gene from the WT strain as expected (2908 + 926 – 1324 = 2350 bp for *rad5*, 2781 + 926 – 1712 = 1995 bp for *rad16A* in WT, 4515 + 926 – 1350 = 4091 bp for *rad16B* in WT, and 27819 + 2832 – 36185 = 1995 bp for *rad16A* in the null mutant of *rad16B*). DM, deletion mutant. CM, complementation mutant. ML, molecular ladder of genomic DNA. (**I–K**) Relative transcript (RT) levels of *rad5, rad16A* and *rad16B* in the 3-day-old SDAY cultures of their mutants versus WT, respectively. (**L**) RT levels of *rad16A* and *rad16B* in the 3-day-old cultures of three double-deletion mutants (DDM1–3) versus WT. Note that the expression of each target gene was not detectable in its DM but well restored in its CM. Error bars: standard deviations of the means from three cDNA samples derived from independent cultures of each strain.

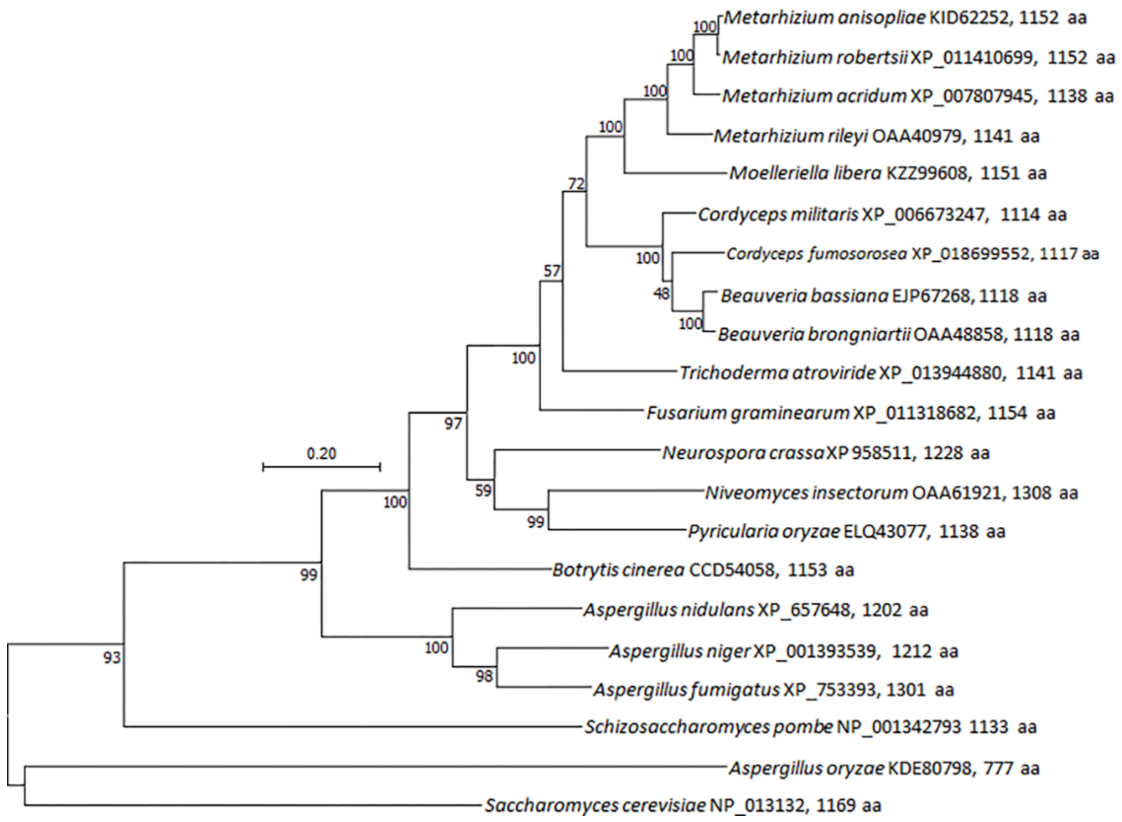

Figure S2. Phylogenetic relationships of Rad5 orthologs found in selected ascomycetes. The maximum likelihood method in the online program MEGA11 at http://www.megasoftware.net/ was used in the analysis. Bootstrap values of 1000 replications are shown at nodes. Scale bar: branch length proportional to genetic distance. Each fungal name is followed by the NCBI accession code of each protein and the length of its amino acid sequence, respectively.

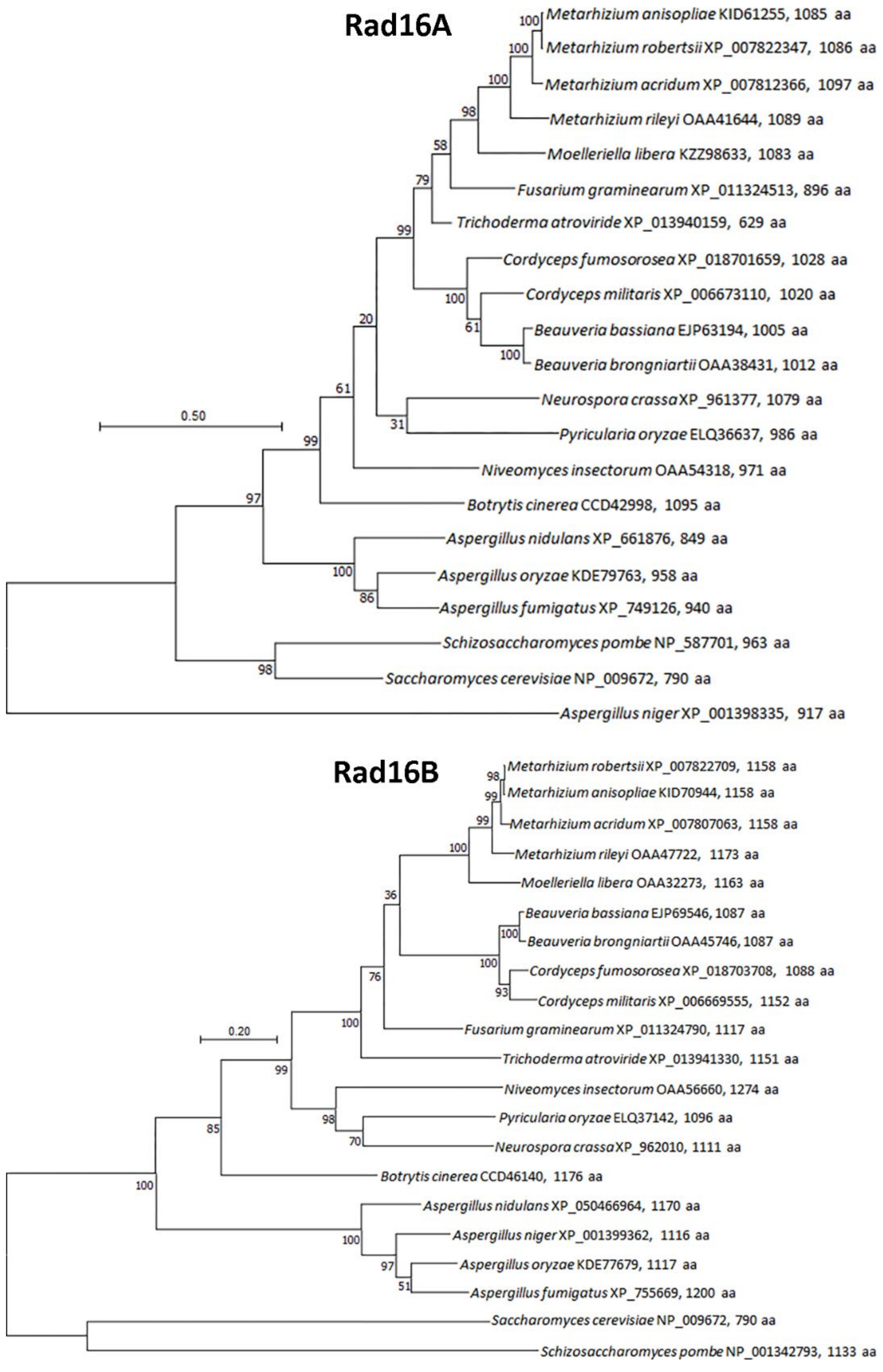

Figure S3. Phylogenetic relationships of Rad16 homologs found in selected ascomycetes. The maximum likelihood method in the online program MEGA11 at http://www.megasoftware.net/ was used in the analysis. Bootstrap values of 1000 replications are shown at nodes. Scale bar: branch length proportional to genetic distance. Each fungal name is followed by the NCBI accession code of each protein and the length of its amino acid sequence, respectively. Note the existence of two Rad16 paralogs (Rad16A and Rad16B) in all the examined fungi except *S. cereviciae*.

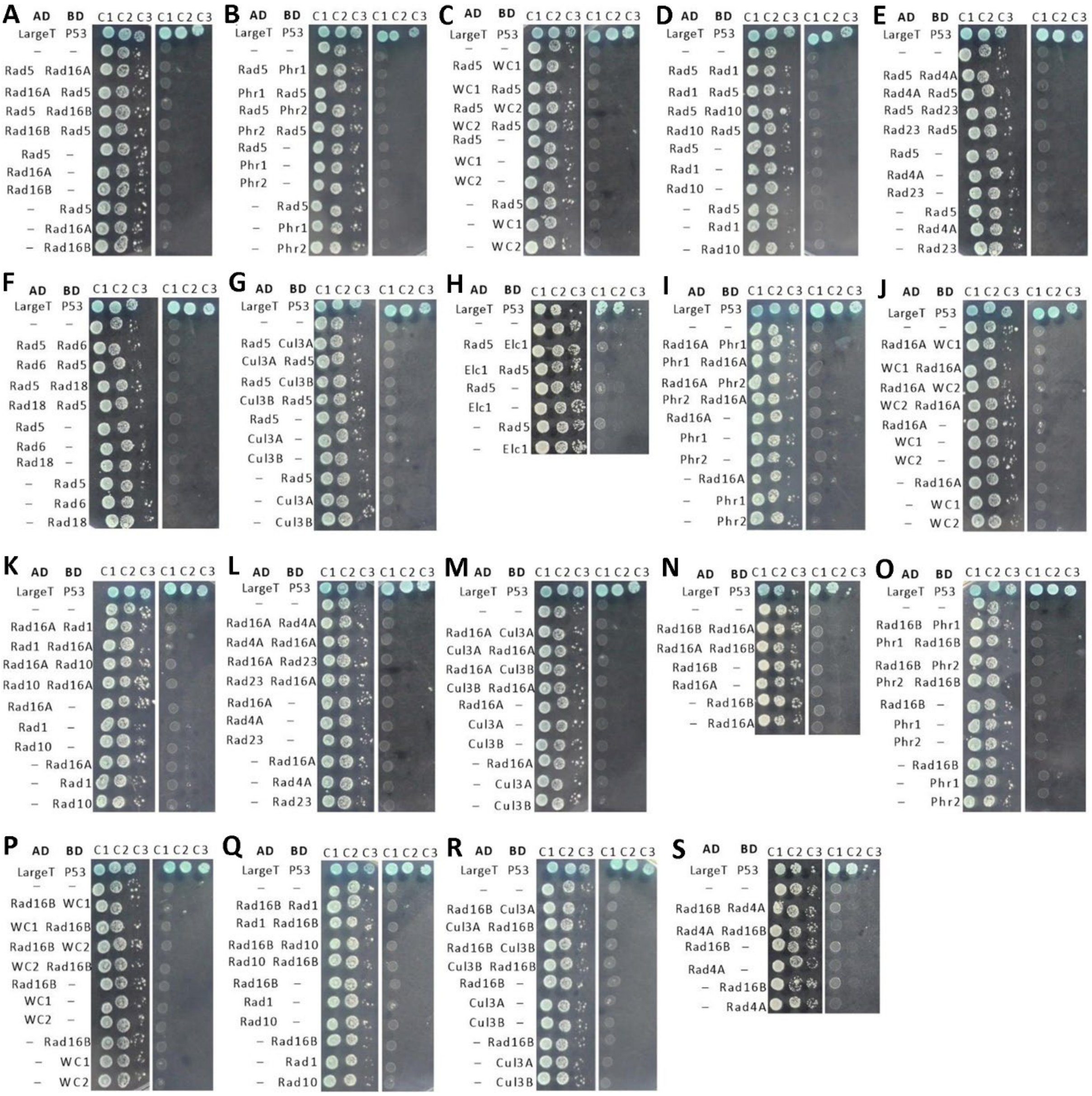

Figure S4. Y2H assays for negative protein-protein interactions in *B. bassiana*. (**A–S**) No signals for interactions of Rad5, Rad16A or Rad16B with photolyases (Phr1 and Phr2), photolyase regulators (WC1 and WC2), Cul3 paralogs (Cul3A/B) and other anti-UV RAD proteins compared to positive control (AD-LargeT-BD-P53) on quadruple-dropout plates (right image in each panel). All yeast colonies initiated with 5×10^4 (C1), 5×10^3 (C2) and 5×10^2 (C3) cells were incubated at 30°C for 3 days.
